# Map Learning with a 3D Printed Interactive Small-Scale Model: Improvement of Space and Text Memorization in Visually Impaired Students

**DOI:** 10.3389/fpsyg.2017.00930

**Published:** 2017-06-09

**Authors:** Stéphanie Giraud, Anke M. Brock, Marc J.-M. Macé, Christophe Jouffrais

**Affiliations:** ^1^IRIT, UMR 5505, CNRSToulouse, France; ^2^IRIT, UMR 5505, University of ToulouseToulouse, France; ^3^Inria Bordeaux, PotiocTalence, France; ^4^Laboratoire Bordelais de Recherche en Informatique, UMR 5800, University of BordeauxTalence, France; ^5^IPAL, UMI 2955, CNRSSingapore, Singapore

**Keywords:** interactive maps, visual impairment, low-cost prototyping, tangible user interfaces, education technology, spatial cognition, special needs

## Abstract

Special education teachers for visually impaired students rely on tools such as raised-line maps (RLMs) to teach spatial knowledge. These tools do not fully and adequately meet the needs of the teachers because they are long to produce, expensive, and not versatile enough to provide rapid updating of the content. For instance, the same RLM can barely be used during different lessons. In addition, those maps do not provide any interactivity, which reduces students’ autonomy. With the emergence of 3D printing and low-cost microcontrollers, it is now easy to design affordable interactive small-scale models (SSMs) which are adapted to the needs of special education teachers. However, no study has previously been conducted to evaluate non-visual learning using interactive SSMs. In collaboration with a specialized teacher, we designed a SSM and a RLM representing the evolution of the geography and history of a fictitious kingdom. The two conditions were compared in a study with 24 visually impaired students regarding the memorization of the spatial layout and historical contents. The study showed that the interactive SSM improved both space and text memorization as compared to the RLM with braille legend. In conclusion, we argue that affordable home-made interactive small scale models can improve learning for visually impaired students. Interestingly, they are adaptable to any teaching situation including students with specific needs.

## Introduction

**FIGURE 1:**
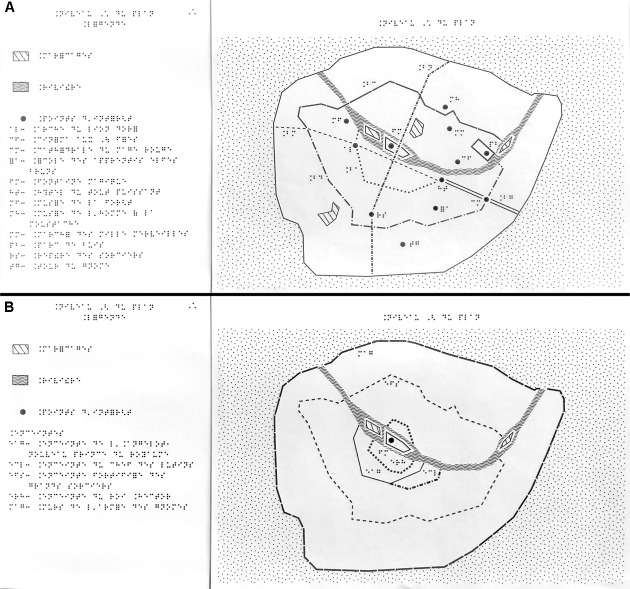
The two raised-line maps (RLM) with their corresponding legends used in the control condition. The first map **(A)** represents the landmarks and roads of the kingdom in its current state as well as the river. The second map **(B)** shows the enlargement of the ramparts over time.

With more than 285 million visually impaired people in the world, including 19 million visually impaired children below the age of 15 (WHO, 2012), it is important to provide adapted and accessible tools that help them understand spatial concepts used in geography, science, and mathematics. Several tools are currently used, such as raised-line maps (RLMs) printed on swell-paper (see **Figure 1**); and numerous studies have demonstrated the benefits of those maps for spatial learning (Ungar, 2000). However, raised lines maps are expensive, cumbersome to produce, and cannot easily be adapted to all learning situations (Rice et al., 2005). In addition, RLMs do not provide any interactivity or flexibility to update the displayed information. Therefore, in the past decades, several researchers have worked on the design of interactive maps for visually impaired people (see Ducasse et al., 2017 for a review). In many prototypes, RLMs are augmented with interactive audio output, so that the user can trigger sound or verbal descriptions when selecting specific items on the map. Recently, several authors have investigated the use of 3D printing for the creation of accessible interactive 2.1D maps (2.1D usually refers to relief with just one height). Götzelmann (2016) and Taylor et al. (2016) provided online platforms that allow to create and 3D print such maps. Printed 2.1D maps can then be laid over a touch-enabled device that adds interactivity. It has been shown that such interactive audio-tactile maps are more usable than regular RLMs with braille legend (Brock et al., 2015). Small-scale models (SSM) are an alternative to RLMs for acquiring spatial knowledge in the absence of vision. Picard and Pry (2009) showed that exploring a SSM of an urban environment improves allocentric spatial learning in visually impaired users. In general, such models are made of wood or plastic and do not provide any interactivity or feedback when they are touched. However, it is now possible to embed different sensors (e.g., buttons or conductive painting) or to use capacitive technologies (Sato et al., 2012) to make those models interactive and provide multisensory feedback.

Tangible interaction has primarily been studied with sighted users, and has been defined as the interaction with a computer through the use of physical objects (Ullmer and Ishii, 2000). Tangible User Interfaces (TUIs) are a bridge between digital and physical worlds, and enhance interaction with digital information (Shaer and Hornecker, 2009). Marshall et al. (2007) proposed a conceptual framework showing all factors that can influence whether and how TUIs might support learning. According to this framework, TUIs provide multiple benefits for learning. First, because body movements and cognition are linked, movements resulting from interaction with a TUI can facilitate cognitive processes, such as spatial cognition. Second, TUIs support collaboration between users. Shared spaces allow users to monitor each other’s gaze, increase the visibility of actions, facilitate increased awareness and situated learning, provide multiple access points for effective turn-taking, and enable users to manipulate physical artifacts outside of the interaction space to improve social organization and planning. Third, TUIs are more accessible or intuitive for certain users groups, especially for children. Fourth, digital information coupling with physical activity may improve reflection in children thanks to the novelty effect. Fifth, TUIs can increase the playfulness of learning. Similarly, Schneider et al. (2011) showed that TUIs enable users to integrate physical and digital representations, stimulate exploratory activities, and improve the quality of thinking. Therefore, when using TUIs at school, students carry out learning tasks with better performance. For instance, high school students explored more options to solve a problem and proposed a better final solution with TUIs than using paper and pen (Do-Lenh et al., 2010). Kwok et al. (2016) also showed that young children (aged 4–8) receiving instructions from an adult in a face-to-face situation reached the same performance when instructions were provided by an interactive tangible device. Moreover, Cuendet and Dillenbourg (2013) showed a good usability of TUIs in the context of apprenticeship. They created a computer environment supporting collaborative learning for carpenter apprentices (“TapaCarp”). After only a few minutes with “TapaCarp,” the students were able to complete complex activities, and judged the device useful and usable. Different studies have shown that increasing the control of the learner over a TUI resulted in better comprehension (Tabbers and de Koeijer, 2010), while reducing cognitive load (Hasler et al., 2007). TUIs are also beneficial for collaborative tasks with children, such as collaborative problem solving (Xie et al., 2008). Interestingly, tangible interfaces also improve the accessibility for young children, people with learning disabilities or novices (Wyeth, 2007; Schneider et al., 2011). Hengeveld et al. (2009) designed interactive construction kits for severely impaired children with cognitive and perceptual-motor disabilities. During the study, they observed longer attention spans and more initiatives from the children. They concluded that the designed TUIs provide access to a rich learning environment with more opportunities for cognitive, linguistic, and social learning than a traditional graphical user interface. They also reported that it encourages collaboration as well as communication behaviors.

Recent studies have demonstrated the benefit of using tangible interfaces in the context of visual impairment. McGookin et al. (2010) designed a TUI that allows visually impaired users to access graphs and charts. They showed that the device improved the accuracy with which participants carried out the tasks. They provided design recommendations for non-visual tangible interaction, such as choosing object shapes that are distinctive by touch alone. Ducasse et al. (2016) demonstrated the usability of “Tangible Reels,” both for the construction and exploration of interactive maps. The results showed that visually impaired users were able to understand and interact with existing drawings, but also to create new ones. Brulé et al. (2016) showed that using tangible objects as part of a multisensory map increased engagement of visually impaired students during learning activities. Using TUIs, visually impaired users can manipulate digital information via physical objects (“phycons”), which involves the sense of touch and is therefore adequate in the absence of vision. In addition, tangible interfaces can provide structural (shape and volume) and material (texture) properties that are important during tactile exploration (Klatzky et al., 1985).

As mentioned above, it has previously been shown that SSMs are beneficial for the acquisition of spatial knowledge by visually impaired people (Picard and Pry, 2009). Yet, to our knowledge, no study has been conducted to evaluate non-visual learning using interactive SSMs. Thus, we aimed to evaluate the usability of an interactive SSM to learn a large and complex geographical and historical display without vision, in comparison to a RLM with braille legend. The interactive SSM and the RLMs used in the current study represented geographical and historical knowledge of a large fictitious kingdom. The interactive small-scale included movable 3D pieces (similar to a puzzle), carved roads in a piece of wood, and verbal descriptions triggered during tactile exploration. The RLMs described the same kingdom with embossed drawings and booklets printed in braille. We set the hypothesis that the interactive SSM is more usable than regular RLMs accompanied by braille legends, i.e., that it allows participants to better memorize geographical and historical information, and that it is more satisfactory to use.

## Materials and Methods

### Participants

We recruited participants from an education center for visually impaired students (INJA in Paris, France). All participants or their legal representatives gave written informed consent to participate. The protocol followed the Declaration of Helsinki and was approved by the CLERIT ethics committee.

Twenty-four blind students (10 females; age range: 14–19 years; *M* = 16.9, *SD* = 1.6) volunteered to participate in this study. All participants were attending High School grades 9–11. They had no other disabilities, and frequently use RLMs during lessons. Before the experiment, we sent a questionnaire to their teacher in order to retrieve the personal characteristics of all participants including age, gender, grade, visual impairment, spatial skills, and braille reading skills. Spatial and braille reading skills were based on the subjective assessment of the teacher.

We divided participants into two groups: the control group explored the RLMs, and the test group test explored the interactive SSM. Within each group of participants, we included five participants with low to medium spatial skills (hereafter called “non-experts”), and seven participants with a good to very good level of spatial skills (called “experts”; see **Table 1**). All participants were proficient braille readers. The control group comprised six females and six males, including three attending grade 9, six attending grade 10, and three attending grade 11. The test group included four females and eight males, including three attending grade 9, seven attending grade 10, and two attending grade 11.

**Table 1:** Number of early and late blind subjects with high (experts) and low (non-experts) spatial skills.

Condition	Blindness	Spatial skills	Number
**RLM**	Early	Experts	5
	Early	Non-Experts	4
	Late	Experts	2
	Late	Non-Experts	1

**SSM**	Early	Experts	4
	Early	Non-Experts	3
	Late	Experts	3
	Late	Non-Experts	2
		Total	24

*RLM, Raised-Line Maps; SSM, Small-Scale Model*.

### Material

We prepared this study in collaboration with a teacher specialized in low-vision education. His objective, beyond the scope of the current study, was to design his own teaching material based on low cost prototyping tools. The spatial layout represented the city where the students are living (Paris, France), in order to be used for giving Geography and Orientation lessons after the study. For the current study, we turned the layout upside down (i.e., North facing 6 o’clock) and created a scenario based on the history of a fictitious kingdom. We checked afterward that none of the students was aware that the model represented the map of the city they lived in. In addition to the specialized teacher, a person in charge of producing tactile documents checked that all materials (RLM, 3D model, braille booklet, text) were appropriate for tactile exploration by the visually impaired students.

The information content of the two conditions (control vs. test) was identical. Two levels of display described the kingdom. The first level showed the main landmarks and roads of the kingdom in its current state, as well as the river going from west to east. The second level illustrated the enlargement of the kingdom with the fortifications that were built over time. The evolution of the kingdom was associated to a complex history^1^.

The control condition encompassed two regular RLMs with a legend (**Figure 1**) and accompanying booklet describing the history of the kingdom printed in braille. The first map corresponded to level 1 of the model (current state of the kingdom) and the second map to level 2 (fortifications). The RLMs were printed on swell paper of the brand ZY^®^-TEX2, and heated with a ZY-Fuse. The legend was printed in braille on two sheets of paper for the first map representing routes and points of interest, and one sheet of paper for the second map representing the ramparts. The legend provided the name of each point of interest. The history of the kingdom was printed in braille in a 20-page booklet for the first level, and an 8-page booklet for the second level. Each booklet provided a short description of each point of interest in the map, in the form of historical and cultural context (e.g., who built it; what it was used for; etc.)

**FIGURE 2:**
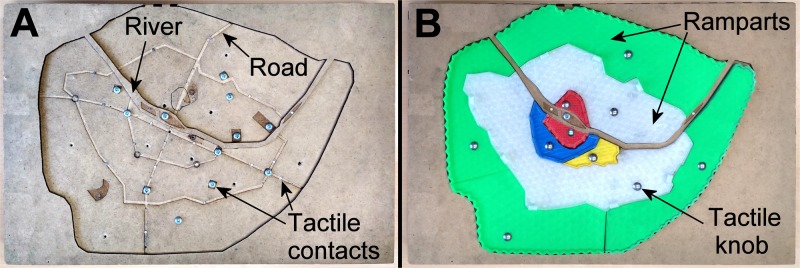
The first level of the interactive small-scale model created with a laser cutter and a wooden plate. This level **(A)** includes the main landmarks and roads as well as the river flowing through the kingdom. Ten plastic 3D printed pieces fit on top of the wooden box. This level **(B)** shows the enlargement of the ramparts over time. Each element is connected with a small metallic knob that triggers verbal descriptions.

The interactive SSM was designed to be affordable and simple to make (**Figure 2**). The wooden box was designed with a vector graphics editor (e.g., Inkscape, Free Software Foundation, Inc.) and produced using a laser cutter. The roads were engraved on top of the wooden box with the same laser cutter. The ramparts were represented with ten 3D printed pieces that fitted on top of the wooden box as a puzzle. The 3D pieces were designed with Blender (Blender Foundation) and printed with a Makerbot printer (MakerBot^®^ Industries). The river was represented by a wooden bulge pasted onto the box. Conductive tactile zones were created on landmarks, ramparts, and roads with small metallic pieces inserted into the wood or plastic pieces, which were connected to two Arduino-compatible Touch Boards^®^ (Bare Conductive Ltd, London). One tactile knob (round head nail) for each rampart and two to five tactile elements (headless nails) for each road triggered audio descriptions when they were touched. Audio feedback included the names of the touched element, as well as the corresponding historical description. Audio files were created with the “Dspeech” (Dimitrios Coustoumbas) software in MP3 format, using the female voice “Virginie” (RealSpeak Solo). The overall cost of the hardware was less than 300€.

Before the experiment, we carried out a set of pretests in order to check that all documents and the prototype were usable. Pretests were completed by three sighted users that were blindfolded, and three visually impaired users that did not participate in the study. The main objectives of the pretests were: (1) assess the duration needed for the complete exploration of the maps; (2) verify the readability of the tactile maps (check that the different symbols and textures were distinguishable and readable), as well as the usability of the 3D model (check that there was no problem in triggering the audio feedback); (3) check that the whole experimental protocol was adapted to visually impaired people, and did not take too long. As a result of the pretests, we improved the prototype (reliability of interactive zones), and set the maximal exploration time during the experiment to 35 min. This duration was sufficient for a person with regular braille reading skills to explore the whole display and read the legends and booklet in both conditions.

### Experimental Procedure

As previously mentioned, participants were included either in the control group (RLMs) or test group (interactive SSM), which corresponds to a between-subject experimental design. Experiments were carried out individually in a quiet and separate room. The experimenter gave instructions, and read the questionnaires after exploration. Magliano et al. (1995) observed that subjects remember different types of spatial knowledge (landmark, route or survey knowledge) depending on the instruction before exploration. Thus, in order to motivate participants to memorize all types of spatial information, we did not provide any cue on the kind of map knowledge that they should retain. In both test and control conditions, participants were free (no specific instructions) to explore the first map level for 10 min; and then the second level for 10 min. Fifteen minutes of additional exploration time was provided to explore the two levels together, resulting in a total exploration time of 35 min.

After exploration, participants were asked to recall the names of all roads, landmarks and ramparts that were present on the map, without receiving any clue from the experimenter. Following free recall, the experimenter read aloud the entire list of the points of interest in order to provide all participants with the same amount of lexical information before the next series of questions. Then, she asked a list of geographical and historical questions about the kingdom. For each question, the subject had to pick the correct answer among four propositions, thus for each question, the probability to answer correctly by chance was 0.25. The presentation order of the geographical and historical questionnaires was counterbalanced across subjects. The order of the questions and of the four choices were also counterbalanced across participants. The last questionnaire aimed to evaluate the subjective satisfaction (SUS) of participants. The whole experiment lasted about 85 min including map exploration.

### Questionnaires

We used two questionnaires to assess the memorization of geographical and historical knowledges. The geographical questionnaire was inspired by previous studies (Kitchin and Jacobson, 1997; Picard and Pry, 2009; Brock et al., 2015). This questionnaire consisted of 24 questions assessing the memorization of routes between landmarks (route knowledge), as well as the spatial layout of the kingdom (survey knowledge). The twelve questions related to route (R) and survey (S) knowledge were each divided into three blocks of four questions.

The three types of route (R) questions concerned: (1) Route distance estimation: Subjects had to select the two landmarks separated by the shortest route among four propositions; (2) Route recognition: Four routes between two points were described. Participants had to pick the correct one; (3) Wayfinding: a starting point, a destination, and the beginning of the route between these two points were provided. Participants had to choose the road that completed the route among four options.

The three types of survey (S) questions were: (1) Direction estimation: a starting point and a goal were given. Participants had to indicate the overall direction to the goal using a clock reference system (e.g., three o’clock for east) among four possible options; (2) Location estimation: a landmark was provided, and subjects had to choose the rampart that covered this landmark among four possible options; (3) Survey distance estimation: Participants had to choose the pair of landmarks that were separated by the longest distance (“as the crow flies”) among four propositions.

The historical questionnaire to evaluate the memorization of historical knowledge about the kingdom was inspired by Giraud and Thérouanne (2010). The questionnaire consisted of 24 questions, one for each landmark, aiming to evaluate text comprehension (see Van Dijk and Kintsch, 1983). Subjects had to choose the correct answer among four propositions.

We also assessed subjective satisfaction for each device using the System Usability Scale (SUS, Brooke, 1996). We replaced the word “cumbersome” by “awkward” in order to make question 8 easier to understand (Bangor et al., 2008). As suggested by Brock et al. (2015), we also changed the sentence “I would imagine that most people would learn to use this product very quickly” to “I think that most visually impaired people would learn to use this product very quickly”.

### Observed Variables and Hypotheses

As already mentioned, we made the general hypothesis that the interactive SSM would provide better learning for both geographical and historical knowledge. The main independent variable was the map type (RLMs vs. interactive SSM).

The dependent variables were the scores in response to geographical and historical questions (with a maximum of 24), and the score to the SUS (with a maximum of 100). We compared these different scores by map in order to test the following specific hypotheses:

• Hypothesis 1: Users get more correct answers to a questionnaire about the geography of the kingdom after exploring the interactive SSM than after exploring the RLMs;
• Hypothesis 2: Users get more correct answers to a questionnaire about the history of the kingdom after exploring the interactive SSM than after exploring the RLMs.
• Hypothesis 3: Users get a higher score to the SUS questionnaire after exploring the interactive SSM than after exploring the RLMs.

## Results

### Effect on Memorization

**FIGURE 3:**
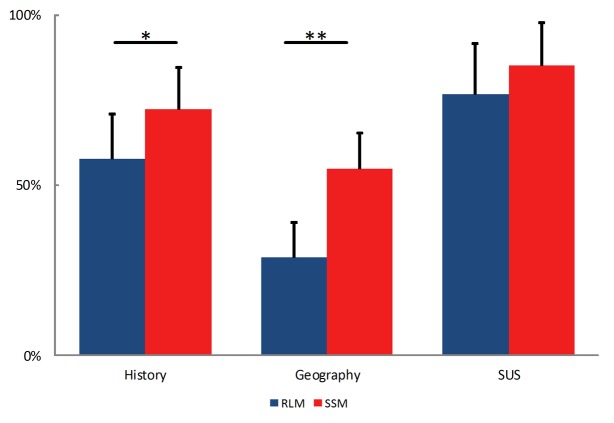
Percentage of correct answers to the historical and geographical questions, as well as SUS score depending on the condition (^∗∗^*p* < 0.001, ^∗^*p* < 0.01).

Shapiro–Wilk test confirmed that each set of data (historical and geographical scores, SUS) was normally distributed. Statistical tests (*t*-test) confirmed our first two hypotheses. Both the correct answers to the historical [*t*(22) = -2.89, *p* < 0.01, η^2^ = 0.27] and geographical questions [*t*(22) = -6.14, *p* < 0.001, η^2^ = 0.63] were significantly higher with the interactive SSM than with the RLMs (see **Figure 3**). **Table 2** presents the mean scores for the historical, geographic and SUS questionnaires.

**Table 2:** Mean scores (*N* = 24; SD in parentheses) to historical questions, geographical questions (route and survey), and SUS for raised-line map and interactive small-scale model.

Dependent variables		Raised-line map	Interactive small-scale model	*t*(22)
**History**		58% (12.7)	72.6% (12)	-2.9^∗^

**Geography**	Overall	28.8% (10.4)	54.9% (10.3)	-6.14^∗∗^
	
	Route	32.6% (16.1)	57.6% (14.4)	-4.01^∗∗^
	
	Survey	25% (12.3)	52.1% (17.5)	-4.39^∗∗^

**SUS**		76.7% (15.1)	85.4% (12.3)	-1.6^ns^

*t-tests between the two conditions are reported in the last column. ^∗∗^*p* < 0.001, ^∗^*p* < 0.01, ^*ns*^*p* > 0.05*.

Among the geography questions, the correct answers to Route [*t*(22) = -4.01, *p* < 0.001, η^2^ = 0.42] and Survey questions [*t*(22) = -4.39, *p* < 0.001, η^2^ = 0.47] were significantly higher with the interactive SSM than with the RLMs. Thus, the interactive SSM improved the memorization of both route and survey knowledge.

**FIGURE 4:**
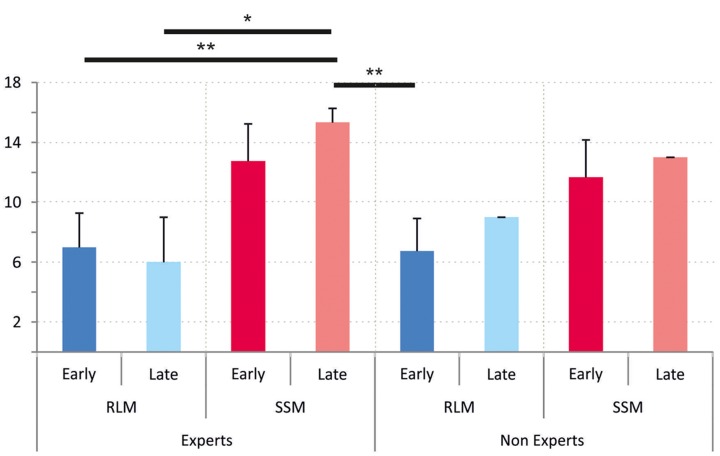
Scores to geographical questions according to onset of blindness and spatial expertise. Error bars represent standard deviation. (RLM, Raised-Line Maps; SSM, Small-Scale Model). Effects of the Tukey *post hoc* pairwise comparisons (^∗^*p* < 0.05, ^∗∗^*p* < 0.01).

It is interesting to note that the enhancement of spatial memorization provided by the SSM affects both the early and late blind, but also experts and non-experts. **Figure 4** breaks down the results by level of expertise and blindness onset. It shows that scores following RLM exploration are between 6 and 8 whereas scores following SSM exploration are between 11 and 15. A 3 factors ANOVA with repeated measures confirmed that only the used tool (RLM vs. SSM) has an effect on spatial memorization score. Both expertise and onset of blindness did not show any significant effect. There was no interaction either. **Figure 4** illustrates that scores are generally better with the SSM than with the RLMs, but the pairwise *post hoc* comparison (Tukey) showed that only late blind using the SSM reached significantly better scores, which is probably due to our limited sample.

We carried out additional analyses on the recall of landmarks (i.e., number of correctly remembered names). The mean scores were 9.2 (*SD* = 3.6) and 10.9 (*SD* = 4.7) for the map and SSM conditions, respectively. There was no significant difference between the two conditions [*t*(22) = -0.99, *p* = 0.33, η^2^ = 0.04].

Finally, there was no significant difference for the SUS scores between the two conditions [*t*(22) = -1.56, *p* = 0.13, η^2^ = 0.10]. Both devices received a good SUS score (RLMs = 76.7; interactive SSM = 85.4). According to Bangor et al. (2008), a device is usable when the SUS score is higher than 70. Thus, both devices were evaluated as usable and satisfactory.

We also checked that results did not depend on spatial skill of the subjects. There was no significant correlation between spatial skills and score for the historical questions [*r* = 0.04, *p* = 0.89, *r*^2^ = 0.002], spatial skills and score for the geographical questions [*r* = 0.002, *p* = 0.99, *r*^2^ = 0.0001]; or spatial skills and SUS score [*r* = 0.27, *p* = 0.40, *r*^2^ = 0.07]. Thus, the interactive SSM had been beneficial for all participants, regardless of spatial skills.

### User Experience

Users explored the interactive map with both hands, i.e., 10 fingers, exactly the same way that they would explore a paper relief map. The interactive map contained no Braille text but audio output was triggered when touching the conductive markers. No further input or output interaction was provided to ensure functional equivalence with the paper map.

We noticed that the participants first explored the content of the whole map with both hands, whether it was the 2D RLMs or the interactive SSM. Then, they typically focused on details. For the 2D RLMs, they explored a landmark on the map, then they moved back and forth between textual and pictorial elements in order to establish the correspondence. On the opposite, when they found a landmark on the interactive SSM, they kept the finger steady on that landmark while listening to the description. In addition, they sometimes moved the second hand to explore the space around that landmark. None of the participants tried to place the first 2D RLM on top of the second 2D RLM in order to make a correspondence between landmarks and ramparts, whereas for the SSM they removed and replaced the 3D pieces and explored which landmarks were under the rampart.

The participants reported that the interactive SSM was much better than the 2D RLMs because of the modularity provided by the 3D pieces, as well as its interactivity. Interactivity was appreciated because then back and forth movements between the map and the braille legend were not necessary. They reported that exploration was easier and faster. They also enjoyed to listen to the verbal descriptions (with earphones when they needed it due to surrounding noise or to focus). However, they declared that the interactive zones were sometimes too sensitive and should be improved.

## Discussion

### Improved Memorization of Spatial and Textual Content When Using Interactive Small-Scale Models

The results show that correct answers to the historical and geographical questionnaires were significantly higher with the interactive SSM than with the RLMs. For the geographical questions, we observed significantly more correct responses for route and survey questions with the interactive SSM. Therefore, the interactive SSM provides an overall benefit for visually impaired students, and the effect size is large. Indeed, 63% of the variance was explained by the type of map for the geography score and 27% of the variance for the history score. These results are consistent with the results of Picard and Pry (2009) which showed that non-interactive SSMs improve the acquisition of spatial knowledge for visually impaired people. However, they differ from those of Brock et al. (2015). Their study aimed to compare the usability of interactive vs. RLMs for visually impaired users. Although they showed that interactive maps are beneficial for visually impaired people in terms of efficiency (decreased learning time) and user satisfaction, they did not observe a better memorization with the interactive map. The discrepancy with the current study can be explained by a ceiling effect in the results obtained by Brock et al. (2015). Indeed, the content of the maps that they used was very simple. It included six points of interest and six streets with straight lines only, whereas the maps that we used in the current study included 1 river, 6 roads, 5 ramparts, 13 landmarks with a complex layout. In addition, the history of the Kingdom was quite long and difficult to memorize, which increased the memory load. Furthermore, in the study by Brock et al. (2015) there was no time limit for exploration, whereas we constrained map exploration to a sufficient but limited time. According to the literature related to gender on spatial cognition, we could expect a difference between males and females in our study. However, it is not possible to balance the factors altogether, especially when working with visually impaired people who are minors. In this study, we focused on a population of young students attending classes in a specialized institute, and we have been able to control the most important factors, i.e., education level, type of visual impairment, and spatial cognition skills. Regarding gender, previous studies showed an advantage of males over females in spatial cognition tasks, but this is controversial as other studies showed no difference (see e.g., Linn and Peterson, 1985; Bosco et al., 2004). Our protocol was not designed to investigate this question further, which is a possible limitation of the study. Therefore, a follow-up study should be conducted to address that specific question.

The SUS score was not significantly better for the interactive SSM, which is notable for two reasons. First, it confirms that visually impaired students were satisfied with the regular RLMs that were made for them, according to their specific needs, relying on the expertise of a person in charge of tactile document making. Indeed, they judged that RLMs were usable with a score of 76.7. Second, it showed that the students have not been sensitive to a “novelty” effect, which may be observed with new devices (Wells et al., 2010). Therefore, it is likely that the significant differences that we observed for geography and history scores are not explained by a stronger motivation associated with the use of the interactive SSM. In addition, this new device seems not to disturb the children’s habits, which is a positive point for classroom teaching.

We did not observe any significant difference between the two conditions regarding the free recall of landmarks. Both devices allowed a good memorization of landmarks, independently of their location. In addition, the recall rate was correlated with history scores for the interactive SSM [*r* = 0.72, *p* < 0.05, *r*^2^ = 0.52]. This correlation shows that good memorization of landmarks also improved text memorization when using the interactive SSM. This is not true for RLMs because the recall rate was not correlated to history scores [*r* = 0.33, *p* > 0.05, *r*^2^ = 0.11]. With RLMs, even though some participants got good recall rates, they did not answer more precisely to the historical questionnaire. Therefore the benefits from the interactive SSM do not rely on a better memorization of landmarks, but rather on a better comprehension of the text.

### Benefits of Interactive Small-Scale Models as Teaching Material

At least two main factors make interactive SSMs an appropriate teaching material for visually impaired students: (1) haptic exploration of spatial content, and (2) multimodal interactivity. First, several authors showed that the haptic sensory system is more efficient when exploring 3D objects than 2D drawings, and hence more efficient to explore SSMs than RLMs in our case. Indeed, in a study by Klatzky et al. (1985) the overall identification process of 3D objects lasted less than 5 s with an efficiency of 96%, whereas it lasted 90 s with efficiency around 30% when 2D drawings were explored. In addition to this low-level perceptual improvement and as mentioned previously, it has been shown that the exploration of SSMs enhances memorization of route and survey in visually impaired users (Picard and Pry, 2009). When interactivity is added to small-scales models, users can benefit from tangible interaction, which helps to better integrate spatial and temporal relationships, but also to stimulate exploratory activities (Schneider et al., 2011). Moreover, it has been shown that multimodality is beneficial for learning (Tindall-Ford et al., 1997). For instance, Dufresne et al. (1995) showed that multimodality (combining auditory and haptic modalities) improved the performance during different manipulation tasks for blind and sighted people. Erhel and Jamet (2011) also showed an improvement of memorization and comprehension, as well as a decrease in perceived cognitive load when using a multimodal interactive device. Using RLMs with a separate braille legend, visually impaired users must integrate information from two separate sources (tactile drawing and legend). The continuous back and forth movements to make the correspondence between textual and pictorial elements disrupts the exploration and divides attention, which results into working memory overload (Sweller, 1999; Erhel and Jamet, 2011). This is not the case with the interactive SSM, where the description of each pictorial element is directly provided by the model itself (Wickens and Andre, 1990).

### Perspectives for Rapid Prototyping of Interactive Small-Scale Models

The teaching material (SSM) that we used in this study was designed in collaboration with a special education teacher. This type of device is relatively easy to design, adapt, and make. In fact, it was mentioned as an initial design recommendation because the teacher wants to be able to produce similar teaching material on his own in the future. First, using the same prototype, it is easy to modify the interactive description associated to each conductive zone. Indeed, it is enough to change the sound files on the micro SD card plugged into the Touchboard. Then, the teacher is free to modify the descriptions according to the perceptual and cognitive abilities of the students, as well as the pedagogical objectives of each lesson. More generally, any professional can design similar interactive teaching materials that fit to her/his own needs (see Giraud and Jouffrais, 2016). Indeed, the decreasing cost of 3D printing and the emergence of easy-to-use electronic boards has recently enabled the development of 3D printed maps (Götzelmann and Pavkovic, 2014; Götzelmann, 2016; Taylor et al., 2016), and physical representations of graphics adapted for children with visual impairment (Kim and Yeh, 2015). “Do-It-Yourself” methods raised a great interest among caregivers to make accessible tactile materials (Stangl et al., 2015). McDonald et al. (2014) created more than ten tactile aids to demonstrate tangible examples of text and web page layouts for visually impaired students using laser cutters and 3D printers. They observed that these aids increased the understanding of the teaching material, but also improved the satisfaction of the visually impaired students. Buehler et al. (2014) also observed the use of 3D printing in three different educational settings involving individuals with cognitive, motor and visual impairment. They observed that 3D design and printing encourage the engagement of the students in STEM (Science, Technology, Engineering, and Math), and allow the creation of accessible teaching material directly by the stakeholders.

Comparing costs for producing these different tools is another important aspect to consider. Teachers probably need 10 to 20 sheets of swell paper for preparing each lesson, depending on the number of failures during the printing process, necessary updates, as well as the number of students. Each pack of 100 sheets costs between 175€ and 330€ depending on the format (A4, A3). In addition to the paper, an inkjet or laser printer as well as a fuser (around 1300€ for ZY-Fuse or PIAF fusers) are needed. The initial cost for producing raised-line graphics is thus important. Obviously, for special education centers that already own a printer and a fuser, the cost is less important. However, although the production of RLMs might seem easy, it requires know-how regarding the design of the map (choice of element sizes, distances, textures, etc.) as well as the handling of the fuser. Then, making a tactile RLM is not an easy task, and is, in general fulfilled by a specialized person called “tactile document maker.” Obviously, 3D printing also relies on dedicated material (3D printer) and know-how. Currently, 3D printers are available at low costs in many cities around the world (thanks to the FabLab movement). But, more importantly, many centers which are specialized in visual impairment have already bought 3D printers and know how to design and print models (this was the case in the school we collaborated with). It is also important to compare the cost and usability of the prototypes designed in this study to the cost and usability of education tools in the market. Very few adapted tools exist and, hence, other tools (e.g., GeoSafari Talking Globe) are presented as educational tools for the blind. In fact this globe has been designed for sighted children and requires the presence of a sighted person to be used. Consequently, it is not adaptable to different usages, children and contents. Its current cost is around 130€.

## Conclusion

We showed that a home-made affordable interactive SSM improves both space and text memorization in visually impaired students. Because such teaching materials are easy to make and affordable, they may cover many different use cases for visually impaired students. Through these technologies, professionals are able to design and build their own teaching material according to their needs (teaching of orientation and mobility, history, geography, mathematics, science, etc.). We are convinced that such accessible, adaptable and low-cost technologies will be accepted and used by special education centers in the future, which will improve education access for visually impaired people.

## Author Contributions

All authors made substantial contributions to the conception of the work and interpretation of data. They all participated in the drafting of the work and approved the version to be published. SG and AB carried out data acquisition, and SG carried out data analysis.

## Conflict of Interest Statement

The authors declare that the research was conducted in the absence of any commercial or financial relationships that could be construed as a potential conflict of interest.

## Footnotes

1 In short, the kingdom first appeared under the reign of king Hector, who died from a poisoned arrow received in the shoulder. After the death of king Hector, the kingdom was enlarged by prince Angelot. Later on, a large rampart was added by the Leader of Sprites. The kingdom was then transformed by the Great Wizards who built the fortifications. The last ramparts were built by the Army of Gnomes around the whole kingdom, which were destroyed during the reunification of all the people. Finally, the kingdom was populated by elves, fairies, gnomes, sprites, humans, wizards, and witches. In total, one river, six roads, five ramparts, and thirteen landmarks (e.g., the forest museum, the gnome tower, etc.) were represented.
